# Ectopic Adipocytes in Scarring Alopecias: Comparison With Normal Scalp and Potential Therapeutic Implications

**DOI:** 10.1111/cup.70131

**Published:** 2026-05-07

**Authors:** Annelise de Almeida Verdolin, Nadia El Kadi, Leonard Sperling, Luciana Pantaleão, Maria Fernanda Reis Gavazzoni Dias

**Affiliations:** ^1^ Department of Dermatopathology Fluminense Federal University Rio de Janeiro Brazil; ^2^ Department of Dermatology Fluminense Federal University Rio de Janeiro Brazil; ^3^ Department of Dermatology Uniformed Services University of the Health Sciences Bethesda Maryland USA

Dear Editor,

1

We recently published an original article showing new histopathological features in cicatricial alopecia [1]. Since normal scalp is not routinely biopsied, a control group was not included in that study. To reinforce our findings, we selected 18 scalp biopsies from patients who complained of hair loss but showed no inflammatory infiltrate or fibrosis in the dermis or follicular regions upon histological examination. These were considered representative of normal scalp tissue and served as our control group. All specimens were obtained with 4 mm punch biopsy. Horizontal sections parallel to the epidermis were performed at 1 mm intervals down to the hypodermis in 31 cases of Frontal fibrosing alopecia (FFA), 29 cases of Fibrosing alopecia in a pattern distribution (FAPD), and 11 cases of Lichen planopilaris (LPP), which were selected based on clinicopathologic and trichoscopic correlation, as well as in 15 cases of normal scalp. Three normal scalp biopsies were sectioned vertically but were included, given that normal scalp biopsies are rarely obtained. All samples were processed and evaluated using deeper serial sections, allowing detailed visualization of the structures.

To better illustrate our findings, we will briefly review normal scalp histology in which the hair follicle is divided into an upper segment (ostium, infundibulum—Figure 1a, and isthmus—Figure 1b) and a lower segment (suprabulbar and bulbar), separated by the bulge zone, where the arrector pili muscle (APM) inserts (Figure 2a). Horizontal sections may show the epidermis and follicle insertion at the ostium and infundibular levels (Figure 1a). The isthmus ranges from the sebaceous duct to the APM attachment at the bulge [2] (Figures 1b and 2a). Secretory eccrine sweat gland coil (ESGC) lies in the dermis below the APM insertion [2] (Figure 2a). Both follicle and ESGC are integrated and embedded in a cone‐shaped portion of dermal white adipose tissue (DWAT), below the APM and sebaceous gland [3] (Figure 2a). Understanding normal scalp histology is essential for the accurate interpretation of histologic alterations and to avoid misinterpretation.

**FIGURE 1:**
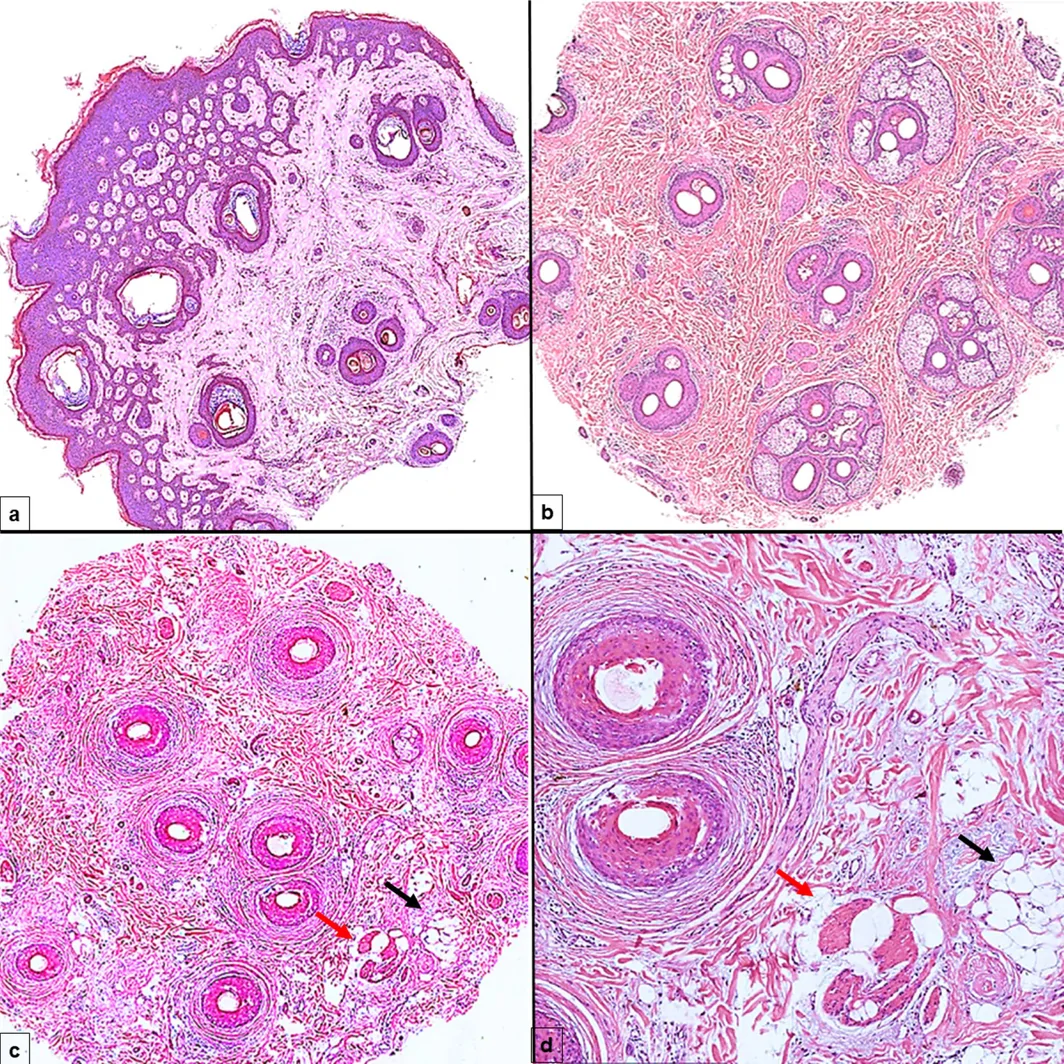
(a and b) Normal scalp. Horizontal section showing the ostium, infundibulum (a) and the isthmus level identified by the presence of the reticular dermis, sebaceous glands, and the arrector pili muscle (b). H&E, original magnification ×100. (c and d) Frontal fibrosing alopecia. This case shows adipocyte infiltration in the dermis at the level of the isthmus (black arrows) and in the arrector pili muscle (red arrows). H&E, original magnification ×100 (c) and ×400 (d).

**FIGURE 2:**
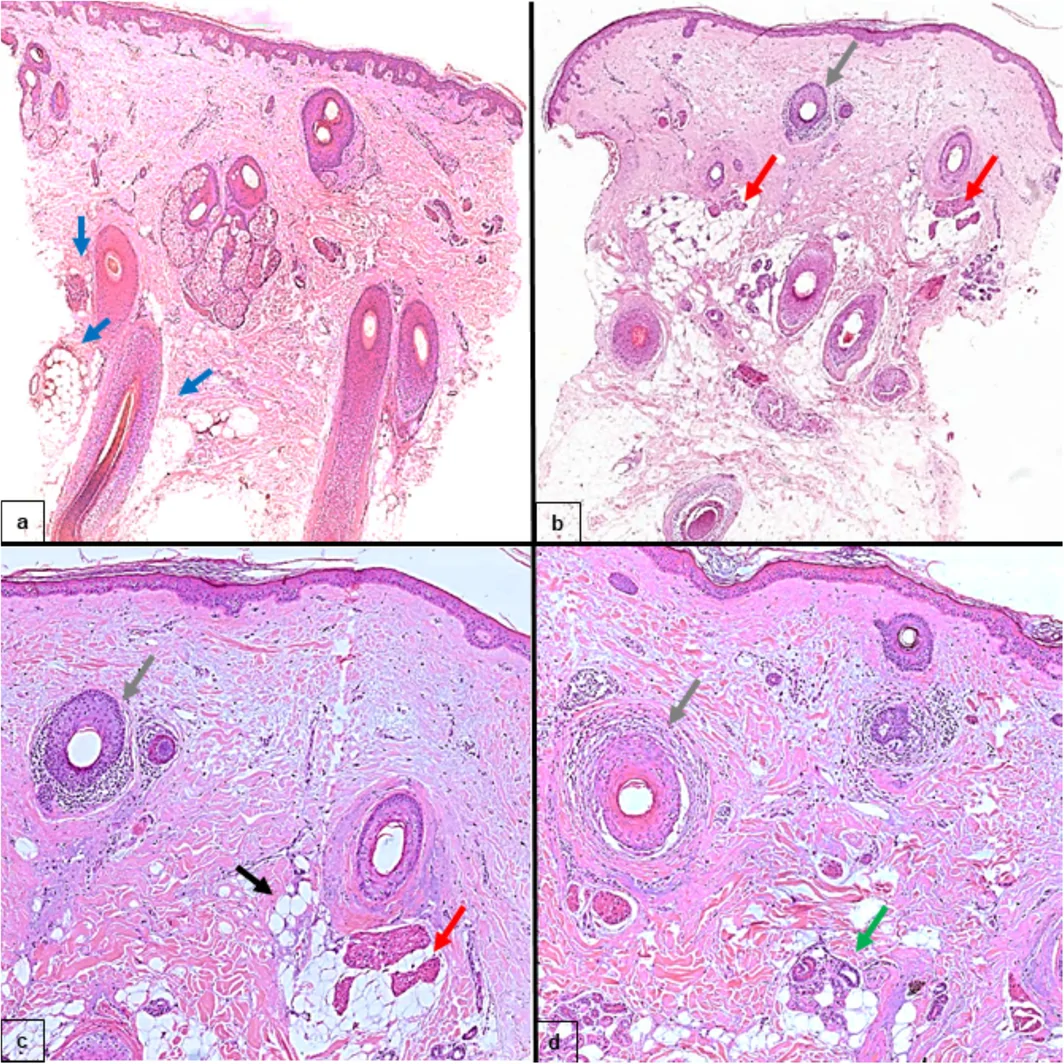
(a) Vertical section of normal scalp. Dermal white adipose tissue surrounds the eccrine sweat gland coil and the lower portion of hair follicle (blue arrows). (b, c, and d) Frontal fibrosing alopecia. There is adipocyte infiltration in the dermis at the level of the isthmus (black arrow) and in the arrector pili muscle (red arrows). Perifollicular lymphocytic infiltrate is present (gray arrows). The eccrine sweat gland coil approaches the epidermis and is at the same level as the arrector pili muscle (green arrow). H&E, original magnification ×200 (a and b) and ×600 (c and d).

In our cicatricial alopecia specimens, we identified adipocytes in the dermis at the isthmus, infiltrating the APM, and ESGC displacement in three scarring alopecias (LPP, FAPD, and FFA) (Figures 1c,d and 2b–d). These changes were first reported by Miteva et al. in FFA cases [2]. We acknowledge that imperfect horizontal sectioning may increase the risk of overinterpreting those findings. To minimize this risk when evaluating the cicatricial alopecia specimens and the control biopsies, we applied the criteria described by Miteva et al. [2] whereby adipocytes at the isthmus level were recorded only when at least three clusters were identified between collagen bundles, in the presence of the proximal insertion of the APM and the level of sebaceous duct entry. Also, isolated adipocytes were not considered in order to avoid confusion with artifacts and dilated vessels [2].

When comparing the control group with FFA, FAPD, and LPP (Table 1), a statistically significant association (*p* < 0.05) was observed between FFA and FAPD and the presence of adipocytes at the isthmus level and/or permeating the APM bundles, and ESGC displacement (Table 1). In contrast, for LPP, only adipocyte infiltration in the dermis at the level of the isthmus showed statistical significance compared with the control group (Table 1).

**TABLE 1:** Comparison of histopathological features between normal scalp (control group) and three scarring alopecias.

Comparison of control group and frontal fibrosing alopecia					
Adipose tissue location	Control group		FFA^a^		*p*‐value of Fisher test
	*n*	%	*n*	%	
*Dermis at the level of the isthmus*					
Absent	17	94.4	6	19.4	< 0.0001
Present	1	5.6	25	80.6	
*APM* ^d^					
Absent	16	88.9	8	25.8	< 0.0001
Present	2	11.1	23	74.2	
*ESGCdisplacement* ^e^					
Absent	17	94.4	20	64.5	
Present	1	5.6	11	35.5	< 0.05
Total	18	100.0	31	100.0	

Comparison of control group and fibrosing alopecia in a pattern of distribution					
Adipose tissue location	Control group		FAPD^b^		*p*‐value of Fisher test
	*n*	%	*n*	%	
*Dermis at the level of the isthmus*					
Absent	17	94.4	9	31.0	< 0.0001
Present	1	5.6	20	69.0	
*APM* ^d^					
Absent	16	88.9	10	34.5	0.0003
Present	2	11.1	19	65.5	
*ESGC displacement* ^e^					
Absent	17	94.4	19	65.5	
Present	1	5.6	10	34.5	< 0.05
Total	18	100.0	29	100.0	

Comparison of control group and lichen planus pilaris					
Adipose tissue location	Control group		LPP^c^		*p*‐value of Fisher test
	*n*	%	*n*	%	
*Dermis at the level of the isthmus*					
Absent	17	94.4	5	45.5	0.005
Present	1	5.6	6	54.5	
*APM* ^d^					
Absent	16	88.9	7	63.6	0.164
Present	2	11.1	4	36.4	
*ESGC displacement* ^e^					
Absent	17	94.4	9	81.8	
Present	1	5.6	2	18.2	0.539
Total	18	100.0	11	100.0	

^a^ FFA, Frontal fibrosing alopecia.

^b^ FAPD, Fibrosing alopecia in a pattern of distribution.

^c^ LPP, Lichen planus pilaris.

^d^ APM, Arrector pili muscle.

^e^ Eccrine sweat gland coil.

Biopsy of normal scalp is rare in our clinic, limiting our sample size. Our 18 control cases offer only preliminary data, and larger studies are needed. The significance of altered DWAT distribution in cicatricial alopecia remains unclear. In our previous study, the presence of adipocytes at the isthmus level was associated with perifollicular inflammation, fibrosis, and fibrous tracts, suggesting a relationship with the inflammatory process [1]. As is well established, DWAT contains adipose tissue–derived stem cells and may play a role in wound healing and skin regeneration; however, it may also stimulate perifollicular fibrosis through adipocyte–myofibroblast transition or inhibit it through the secretion of adiponectin, which has anti‐inflammatory and antifibrotic properties [1]. Poblet et al. also demonstrated the morphological integration of DWAT, ESGC, and the pilosebaceous unit, suggesting these components may influence each other in homeostatic disturbances [3]. Taken together, these findings suggest that adipocytes may play a modulatory role in the inflammatory process in the setting of disrupted local homeostasis.

Studying adipocytes in scarring alopecias may offer insights into disease progression. Peroxisome proliferator‐activated receptors (PPAR *α*, *γ*, *β*/*δ*) are nuclear transcription factors highly expressed in adipocytes and sebocytes that regulate genes linked to inflammation and lipid metabolism (adipogenesis and lipid storage). Defective PPARγ signaling is implicated in pilosebaceous damage, inflammation, and the pathogenesis of scarring alopecias, especially LPP [4, 5]. Although PPARγ may predispose to primary cicatricial alopecias—and agonists like pioglitazone show therapeutic promise—it is unlikely to be the sole factor. Other proposed mechanisms include stem cell loss, immune dysregulation, autoimmunity, and sebaceous gland dysfunction [5].

Investigating the role of adipocytes in abnormal locations in scarring alopecias is necessary to clarify their contribution to inflammation and fibrosis. This could improve our understanding of the pathophysiology of the disease and support the development of new treatment options.

## Funding

The authors have nothing to report.

## Conflicts of Interest

The authors declare no conflicts of interest.

## Data Availability

The data that support the findings of this study are available from the corresponding author upon reasonable request.
